# Research Progress and Prospects on Microbial Response and Gas Potential in the Coal Gasification Process

**DOI:** 10.3390/microorganisms11051293

**Published:** 2023-05-16

**Authors:** Yang Li, Shuheng Tang, Jian Chen, Zhaodong Xi

**Affiliations:** 1 School of Earth and Environment, Anhui University of Science and Technology, Huainan 232001, China; 2 The Key Laboratory of Universities in Anhui Province for Prevention of Mine Geological Disasters, Anhui University of Science and Technology, Huainan 232001, China; 3 State Key Laboratory of Petroleum Resources and Prospecting, China University of Petroleum, Beijing 102249, China; 4 School of Energy Resource, China University of Geosciences, Beijing 100083, China; 5 Key Laboratory of Marine Reservoir Evolution and Hydrocarbon Enrichment Mechanism, Ministry of Education, Beijing 100083, China; 6 Key Laboratory of Strategy Evaluation for Shale Gas, Ministry of Land and Resources, Beijing 100083, China

**Keywords:** coalbed methane, methanogens, biogeochemistry, in situ coal reservoir

## Abstract

As an essential unconventional natural gas resource, China’s coalbed methane resources are only commercially exploited in a few areas, such as the Qinshui Basin and the Ordos. The rise of coalbed methane bioengineering makes it possible to realize the conversion and utilization of carbon dioxide through microbial action and the carbon cycle. According to the metabolic behavior of the underground microbial community, if the coal reservoir is modified, it may stimulate the microorganism to continuously produce biomethane to prolong the production life of depleted coalbed methane wells. This paper systematically discusses the microbial response to promoting microbial metabolism by nutrients (microbial stimulation), introducing exogenous microorganisms or domestication of in situ microorganisms (microbial enhancement), pretreating coal to change its physical or chemical properties to improve bioavailability, and improving environmental conditions. However, many problems must be solved before commercialization. The whole coal reservoir is regarded as a giant anaerobic fermentation system. Some issues still need to be solved during the implementation of coalbed methane bioengineering. Firstly, the metabolic mechanism of methanogenic microorganisms should be clarified. Secondly, it is urgent to study the optimization of high-efficiency hydrolysis bacteria and nutrient solutions in coal seams. Finally, the research on the underground microbial community ecosystem and biogeochemical cycle mechanism must be improved. The study provides a unique theory for the sustainable development of unconventional natural gas resources. Furthermore, it provides a scientific basis for realizing the carbon dioxide reuse and carbon element cycle in coalbed methane reservoirs.

## 1. Introduction

Microorganisms are one of the main driving forces of the cycle of carbon and other life elements in the underground environment. Microbial action has also affected the earth’s supergene system’s environmental change and material cycle, profoundly changing evolution [1]. The commercial development of unconventional natural gas and the clean utilization of coal resources have promoted people’s research on the biomethane production process in coal reservoirs. The rise of CBM (coalbed methane) bioengineering makes it possible to realize the conversion and utilization of carbon dioxide through microbial action and the carbon cycle [2]. In many cases, microbially enhanced CBM can be used as an environmental and economical energy source instead of coal due to the unavailability of most coal resources and the extension of CBM’s well-productive lifespans [3].

Continuous and active microbiology generally exists in coal reservoirs. Biogenic CBM in shallow coal seams is produced from methanogenic microbial communities triggered by surface runoff and meteoric water. However, under natural conditions, the amount of methane produced by the anaerobic fermentation of coal is tiny. There is potential to realize coal biotransformation through manual intervention in the reservoir without biomethane [4,5]. In extremely anaerobic coal reservoir environments, the microbial communities have unique genetic makeup and physiological function relative to the species in conventional conditions. According to the metabolic behavior of the underground microbial community, if the coal reservoir is modified, it may stimulate the microorganism to continuously produce biomethane to prolong the production life of depleted CBM wells [6].

Microbial metabolism of coal into CBM is a multi-step process involving the joint action of microbial communities such as hydrolytic bacteria and methanogenic archaea. The organic macromolecular structure of coal is decomposed into soluble organic substances (long-chain fatty acids, low molecular weight aromatics, alkanes, etc.) by bacteria. Available substrates (CO_2_/H_2_, acetate, etc.) are used for microbial methanogenesis [7]. Aerobic or anaerobic bacteria solubilize and degrade organic components in coal. Methanogens are strictly anaerobic archaea that widely exist in the underground environment. In situ reservoir, physical and chemical factors, and available organic ingredients control their distribution and activity. Studies on the composition of microbial communities in CBM reservoirs worldwide have identified numerous combinations of bacteria and archaea [8].

At present, the proposed microbial means to increase production need to inject beneficial solutions to stimulate the metabolisms of microbial communities. Moreover, it has been identified that the domestication of in situ microorganisms and the introduction of exogenous microorganisms in laboratory environments are feasible, which results in these methods improving the environmental adaptability of these microorganisms [2,8]. In addition, the environmental factors affecting microbial liveness have been optimized. Many studies have focused on the continuous degradation of these critical intermediates. The heterogeneity, complex structure, and high aromaticity of coal determine that the biotransformation of coal is a prolonged process. The low bioavailability of coal is an essential factor limiting its biotransformation [9]. Therefore, coal pretreated by physicochemical means is another important research direction.

The main goal of this study is focused on the analysis of microbial response to stimulating microbial metabolism by nutrients (microbial stimulation), introducing exogenous microorganisms or domestication of in situ microorganisms (microbial enhancement), pretreating coal to change its physical or chemical properties to improve the bioavailability, and improving environmental conditions. This paper systematically combs these essential research directions and puts forward feasible suggestions from laboratory-scale microbial research to its industrial application.

The achievement of the dual carbon goal and the large-scale commercial development of coalbed methane urgently require new technologies. The critical technologies for reducing low or negative carbon emissions in the development of surface coalbed methane and goaf coalbed methane based on coalbed methane bioengineering are of great significance [2,10]. Using CO_2_ and microbial fermentation liquid as working fluids for reservoir transformation achieves reservoir transformation and multiple production increase effects such as CO_2_ bio-methanation, gas boosting, and reservoir modification, providing a new way to increase coalbed methane production and achieving the goal of low-carbon emission reduction. For the development of coalbed methane, CO_2_ can promote its production increase. For CO_2_ storage or conversion, coal reservoirs are one of the best destinations [3,8].

## 2. Characteristics of Microbial Community Structure in CBM Reservoirs

### 2.1. Abundance and Diversity of Microbial Community in CBM Reservoirs In Situ

Microorganisms, including fermentation and sulfate-reducing bacteria, can degrade aromatic compounds and cellulose in coal reservoirs, which are the main types of bacteria driving the biodegradation of coal. For example, *Proteobacteria* is the primary bacterial type in water, coal, and rock samples. However, the bacteria in coal and rock samples are more similar at the genus level. The microbial diversity in the gas sample is significantly lower than in other samples [1,5]. *Spirochaetes* degrade organic substances to produce ethanol, acetate, lactic acid, carbon dioxide, and hydrogen. *Bacteroidetes* are mainly engaged in the degradation of cellulose to produce formic acid, carbon dioxide, and hydrogen. The abundance of *Spirochaetes* increases with the increase in coal rank, but the abundance distribution of *Bacteroidetes* is the opposite. *Firmicutes* are mainly involved in producing intermediates such as acids and alcohols [2]. Significant differences exist in the bacterial community structure in the fermentation system of different coal reservoirs. According to the various substrates available, methanogens can be divided into hydrogenotrophic, acetoclastic, and methylotrophic methanogens [8]. *Rheinheimera* and *Hydrogenophaga* are the main anaerobic fermentation bacteria in coal and water samples from Hubei Province’s high-rank coal mine, respectively. *Methanosaeta* and *Methanosarcina* are the main archaea types in coal and water samples, respectively. The acetoclastic type is the primary biomethane production type, and there may exist hydrogenotrophic and methylotrophic methane production types [6].

Microbial activities and the availability of organic matter determine the rate and degree of coal conversion to CBM in coal reservoirs. In multi-seam coal reservoirs, the lithology segmentation results in infiltration segmentation, water enrichment, and control, affecting microbial activity abundance, diversity, and metabolic intensity [9]. Hydrogenotrophic methanogens dominate the coal-bearing strata in the Cherokee Basin of the United States. The variety of archaea is significantly related to reservoir water solute content. Human activities can affect the biomethane production pathway by affecting the solute of reservoir water, and bacterial diversity has a strong correlation with geographical location, which may result from spatial changes in coal seam maturity [11].

### 2.2. Characteristics of Microbial Community in Laboratory Conditions

Some studies used reservoir water as the microbial inoculation source to degrade coal to produce methane in a suitable laboratory environment (temperature, salinity, pH, etc.) [12]. Yang et al. used the coal seam water of the Qinshui Basin as the microbial inoculation source to cultivate massive anthracite in a large capacity fermentor (160 L), and the composition of bacteria and archaea communities after enrichment and culture is quite different from that under the in situ environment. The main methanogens in the in situ reservoir and enrichment culture are hydrogenotrophic *Methanocalculus* and acetoclastic *Methanosarcinales* [13]. Therefore, the type of methanogens after the enrichment culture cannot determine the biogenic methanogenic pathway in the in situ reservoirs. The study of bacteria, archaea, and fungi in the reservoir water in the Qinshui Basin reported that *Ascomycota* and *Basidiomycota* are the main types of fungi. Fungi play an essential role in the biodegradation of coal, and there is a symbiotic relationship between fungi and methanogens in coal degradation. Using antibiotics to inhibit bacterial activity, facultative anaerobic fungi can co-exist with methanogens. Relatively stable fungal community and volatile fatty acid content are conducive to hydrogenotrophic methanogenesis [14]. Some experimental results show that the microbial community’s diversity decreases, indicating that specific organisms are enhanced under laboratory conditions [15].

He et al. tracked and studied the metabolic process of microbial communities through laboratory culture. During the culture process, the structure of archaea changed significantly, and the kinds of archaea in coal samples and water samples were similar, indicating that the source of samples had limited influence on the distribution of archaea. Due to the diverse metabolic pathways of bacteria, the structure of bacterial flora is determined by the culture stage and affected by the source of samples. The dynamic change degree of the bacterial flora structure is significantly higher than that of the archaea [16]. With different substrates, it was found that the Luling coal field has the potential for hydrogenotrophic, acetoclastic, and methylotrophic methane production. The microbial community changes significantly during enrichment and culture [17]. Therefore, the abundance and energy of different microorganisms affected by induction factors in the culture stage may shift favorably to metabolic processes. Taking the reservoir water of the South Sumatra Basin in Indonesia as the microbial source, the degradation process of Burung sub-bituminous coal, Mangus sub-bituminous coal, and Mangus anthracite and the dynamic change in microbial community structure can be observed. During the enrichment culture, the abundance of bacteria decreased, and the abundance of archaea increased. The abundance change in methanogens corresponds to methane production. Although the microbial communities in the same reservoir are similar, lower-rank coal can produce more biomethane than higher-rank coal [18].

Based on the above research, coal reservoirs contain rich microbial species. Bacteria are more present on the surface of coal pores and fissure structures, while methanogens generally exist in aquatic environments. Some fungi play an essential role in the degradation of organic matter. These microorganisms can degrade coal until methane is produced [19]. Although the microbial community of the coal reservoir has extensive similarities, it has its unique micro biological abundance type and dominant flora. Still, it can accelerate the carbon cycle process by artificially modifying the reservoir environment to induce the enhanced metabolism of specific flora [20].

## 3. Microbial Means Apply for Microbial Communities and CBM Production Potential

### 3.1. Response of Microbial Communities and CBM Potential to Microbial Stimulations

According to the existing research, the research direction of microbial action to increase CBM can be divided into microbial stimulation, microbial enhancement, and physical and chemical ways to improve the bioavailability of coal. These methods can be used alone or in combination to realize the continuous generation of CBM [21]. Coal is a highly heterogeneous organic matter whose chemical composition cannot be accurately characterized. Bacteria preferentially degrade unstable components, convert them into intermediates such as volatile fatty acids, and finally degrade them into usable methanogen substrates. The conversion of coal macromolecules into intermediates is usually a speed-limiting process, and the transformation of these critical intermediates has been the focus of many studies [22].

The method of microbial stimulation refers to adding metabolic intermediates (such as CO_2_/H_2_, acetate, etc.), nutrients (such as nitrogen, phosphorus, etc.), and trace elements (such as trace metal elements, etc.) to activate the activity of microbial communities [23]. The in situ reservoir of a mine in Hubei Province is dominated by thermogenic gas. Still, there are highly diverse microbial types, such as bacteria types such as *Actinobacteria*, *Bacteroidetes*, *Firmicutes*, and *Proteobacteria*, and various acetoclastic, hydrogenotrophic, and methylotrophic methanogens. The addition of acetate can stimulate biomethane production, the abundance of anaerobic bacteria such as *Clostridiales* and methanogens such as acetoclastic *Methanosarcina* increases, and the thermogenic CBM reservoir has the potential to stimulate microorganisms to produce biomethane [6]. Microbial methanogenesis experiments were carried out with volatile fatty acids (sodium acetate, sodium propionate, and sodium butyrate) as organic matter sources from reservoir water inoculated in Erlian Basin and Hailar Basin in Inner Mongolia. The type of bacterial community is closely related to organic substrates. Sulfate-reducing bacteria *Desulfovibrio*, propionic acid oxidizing bacteria *Syntrophobacter*, and butyric acid bacteria *Syntrophomonas* are enriched in sodium acetate, sodium propionate, and sodium butyrate culture solutions, respectively. Methanogens can directly use acetate. Propionic acid and butyric acid require the combined metabolism of bacteria and archaea to produce methane [24]. Thermogenic CBM dominates the south of the Qinshui Basin, but microbial communities’ metabolic activities are in situ. Methanogens are mainly hydrogenotrophic *Methanobacterium*. The enrichment of culture by adding CO_2_/H_2_ and formate confirms the potential of methanogenesis [25].

Nutrients and acetate were added to the in situ bituminous coal reservoir in New South Wales, Australia, and the microbial metabolic response was tracked and studied within 25 months. Microbial abundance increased rapidly in the first 7 months, while acetate consumption and methane production gradually became apparent after 12–19 months. Even if nutrients and acetate stimulate microbial metabolism and the microbial community converts acetate into methane, methane production stops after acetate is exhausted. Even if the microbial abundance increases significantly, methane production activity cannot continue if acetate is not added. About 25% of acetate is converted to methane, indicating the existence of acetate oxidation [26]. Adding intermediates such as acetate can stimulate methane production. However, it must be clear that additives should improve the efficiency of coal macromolecule degradation, not only by providing methanogens with more accessible metabolizing substrates [23].

The content of nutrients such as nitrogen and phosphorus and essential basic nutrients for microbial metabolism such as trace elements may be limited in the in situ environments. The nutrient combination of nitrogen, phosphorus, and other trace elements can promote microbial metabolism and coal degradation [27]. Adding yeast extract, peptone, glutamic acid, amino acids, vitamins, algae extract, and other nutrients can increase methane production to varying degrees [28]. The total methane production and methane production rate increases at an appropriate concentration in the microbial culture test of algae and yeast extracts. The increase in methane production after adding nutrients indicates that the metabolism of underground microbial communities has been strengthened, and relevant extracts such as algae can effectively promote biomethane production. Alternative compound nutrients such as cyanobacteria and yeast can be used as nutritional substitutes to enhance the biotransformation of coal on a large scale [29].

Fe is an essential component of critical functional proteins in anaerobic fermentation bacteria metabolism. Fe usually exists in the form of Fe^0^, Fe^2+^, or Fe^3+^. Fe^2+^ is an integral component of hydrogenase. Hydrogenotrophic methanogens use H^+^ as an electron donor to produce methane with CO_2_ under the action of hydrogenase. The surface of Fe^0^ in an anaerobic environment easily forms a passive microbial film under the action of microorganisms, which seriously weakens the reduction in Fe^0^ and reduces methane production. Fe^2+^ can effectively control the content of H_2_S, remove sulfide through precipitation, and prevent toxic and side effects caused by excessive sulfide accumulation. The presence of Fe^3+^ leads to the transfer of electrons from methane generation to an iron reduction reaction, which inhibits the metabolism and activity of methanogens [30,31]. Adding Fe^2+^ to the microbial fermentation system is conducive to synthesizing hydrogenase, promoting the biomethane production reaction, and it can be used by sulfate-reducing bacteria [32].

Yang et al. used lignite from Shenhua Shengli Coalfield in Xilinhot, Inner Mongolia, to stimulate methanogenic microbial communities with different ethanol concentrations. Under different concentrations of ethanol, the abundance of bacteria increased, but the diversity changed little. The abundance of *Methanobacterium* increased significantly, and the methane production type gradually transitioned to a hydrogenotrophic kind [30]. Ethanol did not vary the bacterial community considerably but strongly affected the archaeal community and methane generation type. The organic source of methane generation comes from coal rather than ethanol. Too much ethanol may inhibit methane generation. In addition to being able to stimulate microorganisms, ethanol is an organic solvent to dissolve small biodegradable molecules in coal. This physical change does not lead to changes in the chemical structure of coal [33,34].

The low hydrogen–carbon ratio in coal is one of the factors limiting the increase in biogas production. Straw is mainly composed of cellulose, hemicellulose, and lignin, with a high hydrogen–carbon ratio [35,36]. The crystal structure of cellulose, the covalent crosslinking between lignin and hemicellulose through ester bonds and ether bonds in plant cell walls, and the complex system of straw make it challenging to biodegrade. The mixed fermentation of straw and coal solves the coal’s hydrogen and carbon ratio imbalance. It optimizes the community structure of bacteria and archaea, which can significantly improve biomethane production. The co-fermentation of coal and straw enhances the efficiency of early organic matter conversion to acetate, CO_2_/H_2_, and methyl compounds. The organic compounds produced by straw degradation activate microorganisms and increase the abundance of beneficial bacteria and archaea for coal biodegradation, which may be the main reason for the synergistic degradation of coal and straw to improve the yield of biomethane [37].

Fermentation bacteria and methanogens were also detected in reservoirs lacking biogenic gas. Microorganisms can remain active in high-order coal reservoirs, but the reservoir environment may not provide sufficient nutrition and metabolic environment for the methanogenic microbial community. Competition and symbiosis among microbial communities are the key factors affecting the structural distribution and the metabolic function of microbial communities. These factors jointly determine the metabolic path and the activity intensity of coalbed fermentation bacteria and methanogens [38].

The production of methane through microbial decomposition of organic components in coal has the characteristics of low cost and low energy consumption. Using biological methods combined with physical, chemical, and engineering techniques in non-minable coal reservoirs with trap conditions can activate microbial metabolism to promote the degradation of organic macromolecules in coal [33,37]. The goal of microbial stimulation is to accelerate microbial metabolic intensity during the degradation of organic matter, providing a usable substrate for methanogens. The metabolism of some methanogens can achieve the recycling and reuse of carbon elements. Nutrient substances such as nitrogen, phosphorus, and iron enter the coal seam as a fracturing fluid, which meets the technical engineering requirements. Replacing compound nutrient solutions with liquid extracts such as algae can reduce costs. Ethanol and other chemical reagents need to be evaluated for feasibility before being applied on site, as chemical reagents may cause damage to the diversity of the reservoir and in situ microbial communities. Straw may damage the crack structure of coal seams and block exhaust ducts, which cannot be promoted on site [36,38].

### 3.2. Microbial Enhancement Accelerating the Process of Coal Microbial Gasification

Microbial enhancement refers to adding/domesticating microorganisms to start or accelerate biomethane production. What is added is a single microorganism with a specific metabolic function or a microbial community that is more active, has more metabolic potential than the current microorganisms and can respond to changes in the external environment. Different microbial community types have been detected in coal seams, shale, and other oil and gas reservoir environments. Microbial metabolic pathways in coal degradation to generate methane may differ in coal seams or even within the same coal seam [39]. Most methanogens are strictly anaerobic, so oxygen may significantly inhibit methanogenesis. The symbiosis of anaerobic and aerobic bacteria in the microbial community can quickly consume oxygen, creating conditions for the growth of anaerobic bacteria to increase the possibility of maintaining their metabolic intensity in different oxygen concentration environments. Denitrification, sulfate reduction, and iron reduction degradation microorganisms provide the metabolic diversity of microbial communities. That is, they respond to changes in the availability of electron receptors and continue to degrade the metabolic activities of coal to maintain the stable production of biomethane. Some methanogens may evolve to cope with a certain oxygen level after natural conditions or artificial domestication, such as *Methanomicrobiales* and *Methanobacteriaes* [23,39].

Biodiversity in nature can be met through the environmental adaptability of microbial communities. Highly competitive microorganisms can quickly adapt to environmental changes and soon become dominant flora. Therefore, some studies have domesticated microorganisms to maximize their metabolic potential [39]. Unable to adapt to the current environment, some microorganisms are iterated or metabolized, while others gradually adapt and eventually form a more targeted microbial community structure [40].

Selected, domesticated, and improved microorganisms can be injected into underground coal seams, and some of the coal can be converted into methane through anaerobic fermentation, achieving the dual goals of increasing coalbed methane production and reducing carbon emissions. In recent years, research on the bioengineering of coalbed methane has mainly focused on the generation pathways of methane from coal anaerobic fermentation, enhanced methane production, and microbial modification of coal reservoirs [23,28,29]. Further research is needed to determine whether the microorganisms domesticated in the laboratory can quickly adapt to underground environments. In addition, it is practical to improve the methane production capacity of the microbial community by introducing exogenous strains, and the addition of exogenous microorganisms should not be based on the principle of disrupting the ecological environment balance. Leading domestic and international experiments generally involve adding nutrient solutions to coal seams to stimulate methane production by native microorganisms in the coal seam [35,37]. There have been no reports of studies on injecting highly efficient microorganisms into underground coal seams after domestication.

## 4. Coal Pretreated by Physicochemical Means and the Response of Microbial Communities

Microorganisms cannot enter coal pores, and their activity scope is limited to the cracks or cleats of coal. Coal reservoirs can expand and extend the coal seam fracture system through fracturing to increase the contact surface between microorganisms and coal [41,42]. Hydraulic fracturing injects mixed fluid into coal seam fractures under high pressure. The chemical reagents added to the fracturing liquid may affect microbial metabolism, and the proppant required for the new fracture maintenance is usually polysaccharide polymer. Microbial sequencing of CBM drainage water in the Sura Basin, Australia shows that the community composition of the hydraulic fracturing wells significantly differs from that of other wells. A close relationship exists between the fracturing fluid’s carbon matrix and the dominant bacterial groups’ metabolism. Fracturing fluid additives are the main reason for the change in community composition [41]. The groundwater flow and oxygen entry increase the abundance and diversity of microorganisms. When a suitable electron acceptor exists, aerobic or anaerobic methane oxidation may consume the coal seam’s residual biogenic and thermogenic methane [43,44].

Decomposing coal into usable substrates for methanogens may be essential in limiting the degree of coal gasification. Using chemical solvents to improve the bioavailability of coal may improve this speed-limiting step, and strong oxidants (such as potassium permanganate or hydrogen peroxide) may help to convert coal into organic acids [45,46]. The microbial community structure and metabolic pathway of sub-bituminous coal in the Powder River Basin after hydrogen peroxide pretreatment show that hydrogen peroxide significantly impacts the microbial system and can dramatically improve the bioavailability of coal [47]. White rot fungi are suitable microorganisms for degrading lignin and can convert polysaccharides into monosaccharides. Compared with other physical and chemical pretreatments, white rot fungus pretreatment has a broader range of applications. Xia et al. pretreated the highly volatile bituminous coal from the Yima coalfield in China with white rot fungi. After pretreatment, the coal surface area and roughness increased, and microorganisms were easier to adsorb on the coal surface. White rot fungi degrade and destroy macromolecules and double-bond groups, forming more carboxyl, hydroxyl, and other oxygen-containing functional groups that are readily biodegradable. After pretreatment, the coal hydrolysis process is significantly shortened, and the gas production cycle is prolonged [48].

Coal’s physical and chemical properties are the main factors limiting its biodegradation. The commonly used laboratory physical pretreatment methods include heating, photooxidation, ultrasonic treatment, high-energy radiation pretreatment, swelling treatment, etc. However, physical processes are challenging for the in situ coal reservoirs. Chemical pretreatment effectively improves coal bioavailability, mainly using oxidants, acids, alkalis, organic solvents, and surfactants to pretreat coal [42,46]. Using strong oxidizing agents such as potassium permanganate to dissolve coal can achieve depolymerization of coal. The common problems with chemical pretreatment are the impact of chemical residues, pH value, and salinity on the environment, so it is necessary to adjust the environment after pretreatment to make it suitable for microbial survival. Hydrogen peroxide is a relatively suitable chemical reagent with little effect on the pH value and salinity and does not introduce foreign chemicals. Pretreatment of coal with strong degrading bacteria can disrupt the structure of coal and improve the efficiency of methane generation [45,47].

## 5. Improve Environmental Conditions and the Response of Microbial Communities

CO_2_ physical storage in coal seams involves coal deformation, fluid migration, adsorption–desorption, and multi-field coupling. Due to CO_2_ dissolution, diffusion, hydrodynamics, and other factors, CO_2_ may be re-released. Hydrogen trophic methanogens can reduce CO_2_ to CH_4_, realizing the carbon cycle and energy conversion [49,50]. Combining microbial action and CO_2_ storage in coal seams can improve CBM production, reduce greenhouse gas emissions, and realize carbon storage and utilization. The application of microbial conversion of CO_2_ to CH_4_ may also be limited by factors such as complex coal seam environment and low bioconversion efficiency. Studying the environmental conditions suitable for microbial community activity is necessary to promote continuous reaction. After CO_2_ is injected into coal seams, methanogens can be activated by substrate induction to realize the conversion of CO_2_ to CH_4_. Most coal seams are in an anoxic environment, and many active or dormant methanogens exist. Microorganisms can degrade and utilize hydrogen in the side chains and functional groups of coal molecules [51,52].

Adding CO_2_ and H_2_ can effectively improve the competitive advantage of hydrogenotrophic methanogens. A low concentration of CO_2_ is conducive to the growth of acetoclastic and methylotrophic methanogens, and the mutual promotion and inhibition of microorganisms is the key to the difference in methane production [51]. The increase in water-soluble CO_2_ or HCO_3_^−^ concentration can ensure the dissolution balance of the storage environment, and most methanogens can use HCO_3_^−^ as an electron acceptor. The biogas production experiment was carried out with bituminous coal and in situ flora in the Qianqiu coal mine, and NaHCO_3_ was added to simulate the CO_2_ storage effect of coal seam water. Methane production shows a low–high–low trend with the increase in HCO_3_^−^ concentration. Microorganisms degrade ether compounds in the early fermentation, and alcohols are degraded later. Although bicarbonate inhibited the growth of some microorganisms, it strengthened microbial the hydrolysis ability of the communities in fermentation and increased the abundance of hydrogenotrophic methanogens. NaHCO_3_ increases the yield of methane and the concentration of organic intermediates [51,52]. Su et al. used the self-designed anaerobic fermentation device to simulate the supercritical CO_2_ environment of in situ coal reservoirs. Methanogens gradually evolved into a single hydrogenotrophic methanogenic mode, significantly increasing biomethane production [53].

The lack of electron acceptors and the low efficiency of electron transfer limit the hydrolysis efficiency of organic matter in the anaerobic fermentation process. The external electric field can improve the degradation rate of organic matter. Hydrogenotrophic methanogens can use CO_2_ to receive electrons at the cathode to reduce methane and simultaneously realize coal gasification and CO_2_ conversion when an electric field is applied on site [54,55]. Zhao et al. have proved that the cumulative yield of methane is higher after using an electric field, and the CO_2_ concentration in the system is significantly reduced due to the conversion to biomethane. The methanogenic pathway changed from acetoclastic to hydrogenotrophic type. *Methanobacterium* tolerates environmental changes and can directly accept electrons to reduce CO_2_ to CH_4_, which may be an important reason for CO_2_ conversion [56]. The external electric field accelerates microbial metabolism and provides ideas for low-carbon or negative-carbon coal gasification [46]. Applying an electric field changes the microbial structure and promotes the extracellular electron transfer and biodegradation of organic matter [57].

The adjustment cost of environmental factors such as in situ coal seam temperature, pH, and pressure is high or impossible. The shallow coal seam can supply microorganisms and nutrients by receiving surface water or atmospheric precipitation input. Artificial modification or selection of a suitable in situ environment is crucial for accelerating the bio-methanation process of coal. Geological storage of CO_2_ provides new ideas for carbon reduction [53,58]. Due to the widespread distribution of unmineable coal seams, it can be used for CO_2_ storage. CO_2_ storage in coal seams is a complex geological process, and various factors, such as the physical properties of coal reservoirs and gas traps, influence its feasibility. There is a risk of CO_2_ leakage in coal seams, and converting it into biomethane through microbial metabolism is an effective way to solve this problem [54,59].

Currently, the geological sequestration of CO_2_ by microbial effect is only in the experimental stage, and many in situ conditions are not considered. Field tests are needed to analyze the efficiency and evolution law of the geological sequestration of CO_2_. At the same time, it is also necessary to combine the optimized response law of the underground microbial community with engineering conditions such as injection and production time to improve the feasibility of CO_2_ storage in coal seams and realize the dual effects of CO_2_ emission reduction and energy regeneration [60,61].

CO_2_ extraction can effectively dissolve the organic matter in coal. The destruction of non-covalent bonds involved in the extraction process leads to the organic matter in small molecules being separated from the macromolecular network of coal and reducing the aromaticity of coal. The pore connectivity of coal becomes improved. The technology’s feasibility, efficiency, and practicability are further enhanced by applying CO_2_ extraction to the biological gasification of coal [56,61]. Especially under supercritical conditions of pieces, the diffusion and solubility of CO_2_ in coal become greatly improved. Supercritical CO_2_ extraction anaerobic fermentation for increasing coal seam gas production is gradually receiving attention. Under ideal conditions, when the burial depth of a coal reservoir exceeds 800 m, the temperature and pressure of the coal reservoir can easily cause CO_2_ to reach a supercritical state [55,59].

## 6. Microbial-Driven Biogeochemical Cycle

The metabolism and evolution of microorganisms are the source power of the geochemical cycle, and they determine the existence form of life elements. With the development of microbial gene sequencing technology and detailed biogeochemical analysis, we have a relatively clear understanding of how microorganisms drive the circulation of substances and elements [2,24]. The content of CH_4_ in the atmosphere has increased sharply since the industrial revolution. The doubling of NH_4_^+^ concentration leads to the deterioration of water quality, and the release of H_2_S poses a significant threat to the ecological environment. Microbial systems in the ecological environment play a crucial role in regulating harmful substances. Microorganisms regulate life elements to balance various natural life element compounds [62,63]. As shown in Table 1, carbonaceous compounds are produced by microorganisms under Gibbs free energy.

Methane is the single-carbon compound with the highest reduction degree. Most of the methane released into the atmosphere is biogenic. The microbial metabolism in various ecosystems controls the methane or carbon cycle. Archaea produce the most biomethane in *Euryarchaeota*, and methanogens are obligatory anaerobes in anaerobic sediments or water bodies. Methanogens are widespread, with CO_2_/H_2_ and methyl compounds as substrates [63,65]. At the order level, most methanogens (including *Methanomicrobiales*, *Methanobacteriaes*, *Methanococcals*, *Methanopyrales*, and *Methanocellales*) can produce methane in a hydrogenotrophic type. *Methanosarcinales* have a wide range of substrates, reducing the methyl group of methanol, methylamine, or methyl thioether to methane. Methanogens with acetate as the substrate are relatively limited to *Methanosarcina* and *Methanosaeta*. Although they have a slower metabolism and low phylogenetic diversity than other methanogens, they produce about two-thirds of the global biomethane [2,65]. According to the previous studies, methanogens are obligatory anaerobic microorganisms, but the metabolic activity of *Candidatus Methanothrix Paradoxum* found in aerobic soil seems to shake the traditional view. *Methanofastidiosa* may be a methanogen connecting the carbon and sulfur cycles, but the lack of amino acid biosynthesis makes it difficult to isolate in pure culture [2,63]. *Methermicoccus shengliensis*, separated from the deep underground environment, can directly use the methoxylated aromatic compounds in lignin, oil, and coal to produce methane. It is essential in the carbon cycle of coal and oil reservoirs rich in underground sediments. *Methermicoccus shengliensis* may be able to degrade coal and produce gas alone, which significantly impacts the formation and utilization of CBM. Methylotrophic methanogenesis involves methyl disproportionation and CO_2_ reduction, and methoxylated substrates (such as methanol) are disproportioned to 3/4 CH_4_ and 1/4 CO_2_, but the microbial metabolic mechanism of methylotrophic methanogenesis is still unknown [2,65]. Therefore, undiscovered methanogens may use more substrates to produce methane in an oxygenated environment, and methanogens may not be limited to *Euryarchaeota*.

**Figure 1:**
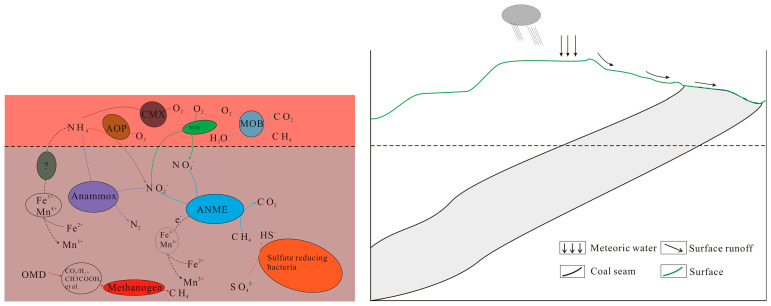
Microbial action in the cycle of carbon compounds in regional coal seams (AOP: ammonium-oxidizing prokaryotes; CMX: comammox bacteria; NOB: nitrite-oxidizing bacteria; MOB: methane-oxidizing bacteria; ANME: anaerobic methane-oxidizing archaea; OMD: organic matter degradation).

Biomethane produced by methanogens can be oxidized by methane *ANME* (*Anaerobic Methanototropicarchaea*) and aerobic oxidizing bacteria, an indispensable part of the global or regional carbon cycle [2,64]. *ANME*, which was discovered first, oxidizes methane with sulfate-reducing bacteria in the seabed methane leakage environment [59]. In marine sediments, the coupling of sulfate reduction and anaerobic methane oxidation is dominated by *ANME* and sulfate-reducing bacteria, and the Gibbs free energy of this reaction is low. In marine and freshwater environments, methane generation and sulfate reduction are mutually exclusive due to the competition for substrates. Too much sulfate may inhibit methanogenesis [2,63]. Compared with sulfate, methane oxidation with NO_3_^−^/NO_2_^−^ as electron acceptors is more in line with the Gibbs free energy law. NO_3_^−^/NO_2_^−^ methane oxidation is widely distributed in wetlands, rivers, marine sediments, and other environments [63]. In addition to SO_4_^2−^ and NO_3_^−^/NO_2_^−^, Fe^3+^ and Mn^4+^ are suitable electron receptors for the anaerobic oxidation of methane, and their microbial mechanism needs further study [2]. There is still a large part of microbial diversity, and its metabolic potential in C, S, and N cycles has not been found [2,63]. Methane not oxidized by anaerobic bacteria can reach the aerobic layer of sediment or soil and be further oxidized by aerobic bacteria [63]. As shown in Figure 1, biomethane formation and methane oxidation may also exist in the underground coal seam environment. Therefore, the synergistic relationship between microbial actions and the underground oxidation–reduction environment should also be considered to generate biomethane in regionally underground environments (such as coal seams).

Microbial solution, nutrient solution, and CO_2_ are injected into underground reservoirs with trap conditions to realize microbial methane alkylation of coal and CO_2_ to obtain low-carbon energy, which has significant carbon emission reduction; ecological environment governance significance provides new development ideas for achieving the goal of carbon neutrality. The adaptation mechanism of microorganisms to the environment is a fundamental scientific proposition in geological microbiology [77,78]. Currently, research on microorganisms in the coal storage layer mainly focuses on describing the response of microorganisms to a single environmental factor under laboratory conditions or the abundance and structure of microbial communities at a sampling point. Further research is urgently needed on the spatial distribution of carbon cycling-related microorganisms at the regional scale, the functions of the ecosystem, and their response characteristics to multiple environmental factors. In addition, the sulfur, nitrogen, iron manganese, and other processes are also coupled through the carbon cycle driven by microbial action. The coupling mechanism between these processes, the carbon element cycle process, and the microbial metabolic characteristics involved are still unclear [79,80]. Therefore, combining in situ engineering geological factors for on-site experiments is urgent.

## 7. Conclusions

The underground dispersed organic matter left over by China’s coal mining, such as goaves and thin coal layers with no industrial development value, provides a rich material source for coal microbial gasification. The implementation of CBM bioengineering is similar to that of conventional CBM development. In the goaf with trap conditions, the evaluation system of gas accumulation in the goaf is first established. Then, the fracturing fluid is transformed into highly efficient bacteria or nutrient fluid. Carbon dioxide is injected into the goaf to gasify dispersed organic matter in residual coal and the surrounding rock, achieving low carbon or even negative carbon effect, which has significant ecological and environmental significance.

The low cost of carbon dioxide methanation by using microorganisms in coal reservoirs under a suitable environment is a prerequisite for its commercial operation. Carbon dioxide bio-methanation requires carbon dioxide, hydrogenotrophic methanogens, and suitable reducing agents. Most of the methanogens in CBM reservoirs are mainly hydrogenotrophic. Hydrogenotrophic methanogen abundance and metabolic intensity can be increased and improved by high-efficiency bacterial and nutrient solutions. Most coal reservoirs are in robust reduction environments; methanogens use HCO_3_^−^ as the primary electron acceptor to produce methane. Therefore, during the implementation of CBM bioengineering, the whole coal reservoir is regarded as a giant anaerobic fermentation system. Coal and carbon dioxide provide a good carbon source for microorganisms, and the coal reservoir offers a suitable environment for the growth and propagation of anaerobic microorganisms. As groundwater migrates and recharges, it also provides electron donors for microorganisms.

The liquid phase material extracted by supercritical carbon dioxide provides substances that are difficult for methanogenic bacteria to degrade under normal conditions. The combined technology of supercritical carbon dioxide extraction and microbial anaerobic fermentation can satisfy the supercritical state of carbon dioxide and be suitable for microbial growth and metabolism. Exploring large-scale, low-cost, commercial underground carbon dioxide storage or reuse technology is a technical problem to solve in low-carbon development.

Furthermore, in the process of using this technology, there are still some theoretical problems that need to be solved. Firstly, the metabolic mechanism of methanogenic microorganisms should be clarified. Secondly, it is urgent to study the optimization of high-efficiency hydrolysis bacteria and nutrient solutions in coal seams. Finally, the research on the underground microbial community ecosystem and biogeochemical cycle mechanism must be improved. It is essential to reveal microbial metabolic function distribution and biogeochemical cycle distribution on the block scale.

## Figures and Tables

**Table 1:** Microbial autotrophic reaction in the process of carbon-containing compounds and microorganisms (ideal environment with a temperature of 25 °C and pH of 7).

Electron Acceptor	Formula	Microorganism	∆G_0_′ (kJ/mol)	Reference
CH_3_OH	4 CH_3_OH → CO_2_ + 3 CH_4_ + 2 H_2_O	*Methanosarcina semesiae*	−103	[64]
CH_3_-R	(CH_3_)_2_SH + H_2_O → 0.5 CO_2_ + 1.5 CH_4_ + H_2_S	*Methanomethylovorans hollandica*	−56	[65]
CH_3_COOH	CH_3_COOH → CO_2_ + CH_4_	*Methanothrix soehngenii*	−36	[66]
CH_3_O-R	4 CH_3_O-R + 2 H_2_O→4 R-OH + CO_2_ + 3 CH_4_	*Methermicoccus shengliensis*	−106	[67]
CH_3_OH	CH_3_OH + H_2_ → CH_4_ + H_2_O	*Methanomassiliicoccus luminyiensis*	−113	[68,69]
		*Candidatus Methanonatronarchaeia*		
		*Candidatus Methanofastidiosa*		
O_2_/H_2_O	CH_4_ + 2 O_2_ → CO_2_ + 2 H_2_O	Methane-oxidizing bacteria (MOB)	−801	[70,71]
		*Alphaproteobacteria*		
		*Methylocella palustris*		
		*Methylocella tundra*		
		*Gammaproteobacteria*		
		*Verrucomicrobia*		
NO_3_^−^/NO_2_^−^	CH_4_ + 4 NO_3_^−^ → CO_2_ + 4 NO_2_^−^ + 2 H_2_O	*Candidatus Methanoperedens nitroreducens*	−503	[72]
NO_2_^−^/N_2_	3 CH_4_ + 8 NO_2_^−^ + 8 H^+^ → 3 CO_2_ + 4 N_2_ + 10 H_2_O	*Candidatus Methylomirabilis oxyfera*	−928	[73]
Fe^3+^/Fe^2+^	CH_4_ + 8 Fe^3+^ + 2 H_2_O → CO_2_ + 8 Fe^2+^ + 8 H^+^	*Candidatus Methanoperedens nitroreducens*, *ANME-2C*	−454	[74,75]
SO_4_^2−^/H_2_S	CH_4_ + SO_4_^2−^ → HCO_3_^−^ + H_2_S + H_2_O	*Anaerobic methanotrophic archaea* (*ANME*)	−21	[76]

## Data Availability

Not applicable.
